# Photoswitchable non-fluorescent thermochromic dye-nanoparticle hybrid probes

**DOI:** 10.1038/srep36417

**Published:** 2016-11-08

**Authors:** Walter N. Harrington, Mwafaq R. Haji, Ekaterina I. Galanzha, Dmitry A. Nedosekin, Zeid A. Nima, Fumiya Watanabe, Anindya Ghosh, Alexandru S. Biris, Vladimir P. Zharov

**Affiliations:** 1 Arkansas Nanomedicine Center, University of Arkansas for Medical Sciences, Little Rock, AR; 2 Center for Integrative Nanotechnology Sciences, University of Arkansas at Little Rock, Little Rock AR; 3 Chemistry Department, University of Arkansas at Little Rock, Little Rock AR.

## Abstract

Photoswitchable fluorescent proteins with controllable light–dark states and spectral shifts in emission in response to light have led to breakthroughs in the study of cell biology. Nevertheless, conventional photoswitching is not applicable for weakly fluorescent proteins and requires UV light with low depth penetration in bio-tissue. Here we introduce a novel concept of photoswitchable hybrid probes consisting of thermochromic dye and absorbing nanoparticles, in which temperature-sensitive light–dark states and spectral shifts in absorption can be switched through controllable photothermal heating of doped nanoparticles. The proof-of-concept is demonstrated through the use of two different types of temperature-sensitive dyes doped with magnetic nanoparticles and reversibly photoswitched by a near-infrared laser. Photoacoustic imaging revealed the high contrast of these probes, which is sufficient for their visualization in cells and deep tissue. Our results suggest that these new photoswitchable multicolour probes can be used for multimodal cellular diagnostics and potentially for magnetic and photothermal therapy.

The development of photoswitchable fluorescent proteins (PFPs) has led to breakthroughs for tracking intracellular proteins and organelles^1^, greatly enhanced super-resolution fluorescent imaging^1^,^2^,^3^,^4^,^5^,^6^,^7^,^8^,^9^ and proven to be effective in tracking single circulating tumour cells^2^. However, conventional photoswitching preferentially utilizes light in the UV and visible spectral range, which only allows for the assessment of the superficial tissue due to strong light attenuation, large autofluorescence and the scattering background of bio-tissue^10^,^11^,^12^. The need to synthesize these proteins using a living vector^13^ complicates the preparation procedures and restricts their use in humans. Little progress has been made in the development of PFPs for near-infrared (NIR) range with deeper light penetration in bio-tissues. Conventional photoswitching is also not applicable for weakly fluorescent proteins.

As an alternative, photothermal (PT) and, especially, photoacoustic (PA) methods demonstrate tremendous potential for noninvasive study of non-fluorescent (most biostructures are weakly fluorescent) proteins, cells, tissue and nanoparticles (NPs) as PT and PA high contrast agents^14^,^15^,^16^. These methods are primary based on the non-radiative relaxation of absorbed energy in targets into heat. Fast thermal expansion of heated zones leads to generation of acoustic waves that are detected by an ultrasound transducer attached to samples^14^,^15^,^16^,^17^,^18^. Because the photoswitching of PFPs leads to spectral shift in both absorption and emission spectra, PT and PA techniques have been applied for spectroscopy and imaging of PFPs before and after switching^19^,^20^. However, relatively low absorption of PFPs prevents broad application of these techniques.

Several photoswitchable NPs with changes in both absorption and fluorescence have been explored for fluorescent switching^6^,^21^,^22^, NP size modulation^23^ and drug delivery^24^. Here we present novel proof-of-concept, photoswitchable non-fluorescent probes as PA and PT contrast agents consisting of thermochromic dye (TCD) and absorbing magnetic NPs (MNPs). The probes’ absorption, and hence PA spectra, is switched upon illumination with NIR laser, creating a photoswitchable system that is responsive in the NIR range.

## Results

### Principle of new photoswitchable TCD-NP hybrid probes

**Figure 1:**
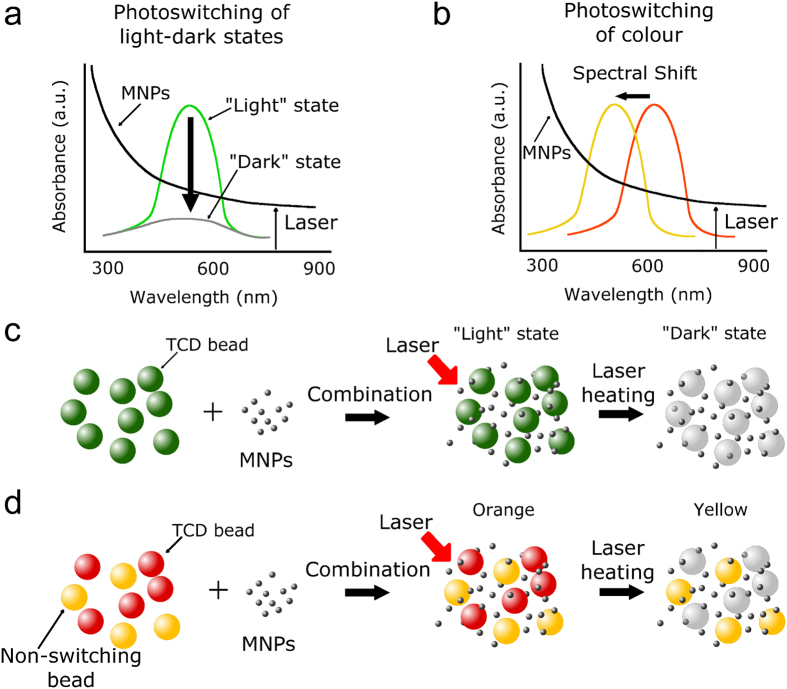
Principle of photoswitchable thermochromic dye (TCD)-NP probes. (**a**) Laser-induced temperature-dependent decrease of TCD absorption (“light” to “dark” state transition) during laser heating of embedded magnetic NPs (MNPs). (**b**) Laser-induced temperature-dependent shift in the TCD absorption maximum (blue-shift colour). (**c**) Schematic of switching from “light” to “dark” state in monocolour TCD-NP probe. (**d**) Schematic of colour in two colour TCD-NP probe.

The principle and schematics of hybrid TCD-NP probes is shown in Fig. 1. We selected two TCDs from LCRHallcrest, LLC^25^, one of which utilizes an absorption decrease schematic, transitioning from coloured “light” state to colourless “dark” state (Fig. 1a) and the other a spectral (blue) shift schematic, transitioning from an orange colour to a yellow colour upon heating (Fig. 1b). TCDs operate through the mechanism of thermochromism, a phenomenon of reversible colour (absorption) change during laser-induced temperature alteration. Because the TCDs absorb light in the visible range, we proposed to use MNPs, which absorb in abroad spectral range, including the NIR (Fig. 1a,b). The relaxation of absorbed energy into heat provides temperature-induced switching of the TCD either from a “light” to “dark” state, or from one colour to another (Fig. 1c,d). Thus, combination of the TCDs with MNPs creates hybrid TCD-NP probes whose absorption can be modulated remotely to either show a decrease in absorption in the case of a homogenously switchable bead system (Fig. 1c) or a spectral shift (e.g., blue shift) in absorption in the case of a heterogeneous mixture of switchable and non-switchable beads (Fig. 1d).

**Figure 2:**
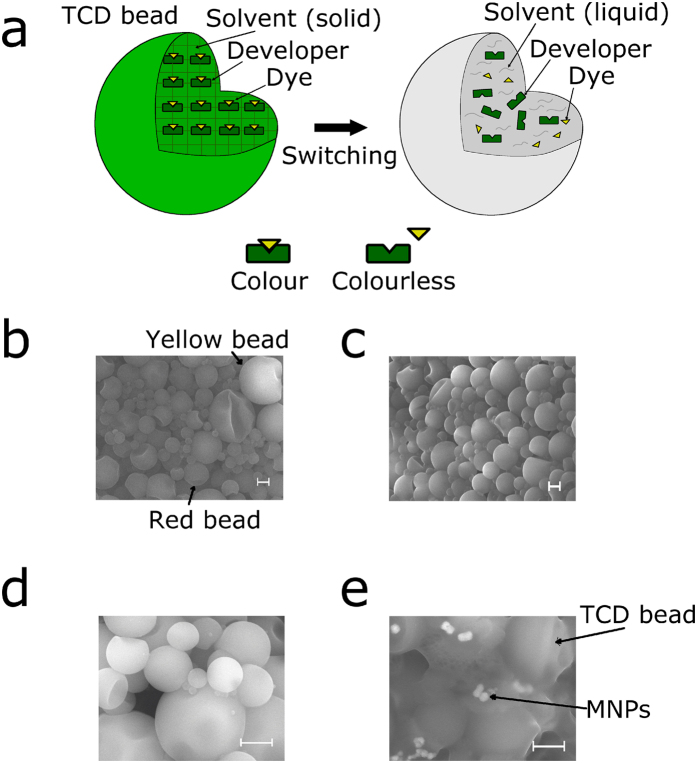
TCD bead structure and SEM images. (**a**) Schematic of a TCD bead. (**b**) SEM image of the orange TCD solution consisting of the yellow and red beads. (**c**) SEM image of red TCD beads alone after filtering out the yellow beads; (**d**) SEM image of green TCD bead solution. (**e**) SEM image of red TCD-NP probes containing red TCD beads with embedded MNPs. (**b**–**e**) Scale bar, 1 μm.

The TCDs used here are microencapsulated chromagenic materials with an impervious polymeric wall made of melamine formaldehyde, ranging from 200 nm to a few μm (Fig. 2)^26^. The mechanism behind the colour change of these specific TCDs is driven by a dye as a colour former, a colour developer (Bisphenol A), and a solvent, all encapsulated by melamine formaldehyde (forming a bead appearance) to prevent oxidation and undesired chemical interactions with the environment (Fig. 2a)^26^,^27^. When the TCD beads are heated, the melting point of the solvent is surpassed and the liquid nature of the solvent frees the developer and dye to interact with other molecules in the medium, changing the chemical nature of the dye and thus switching from a coloured state to a colourless state^26^. Once the TCD beads cool, the solvent solidifies, causing the dye and developer to interact once again, rescuing the original colour.

**Figure 3:**
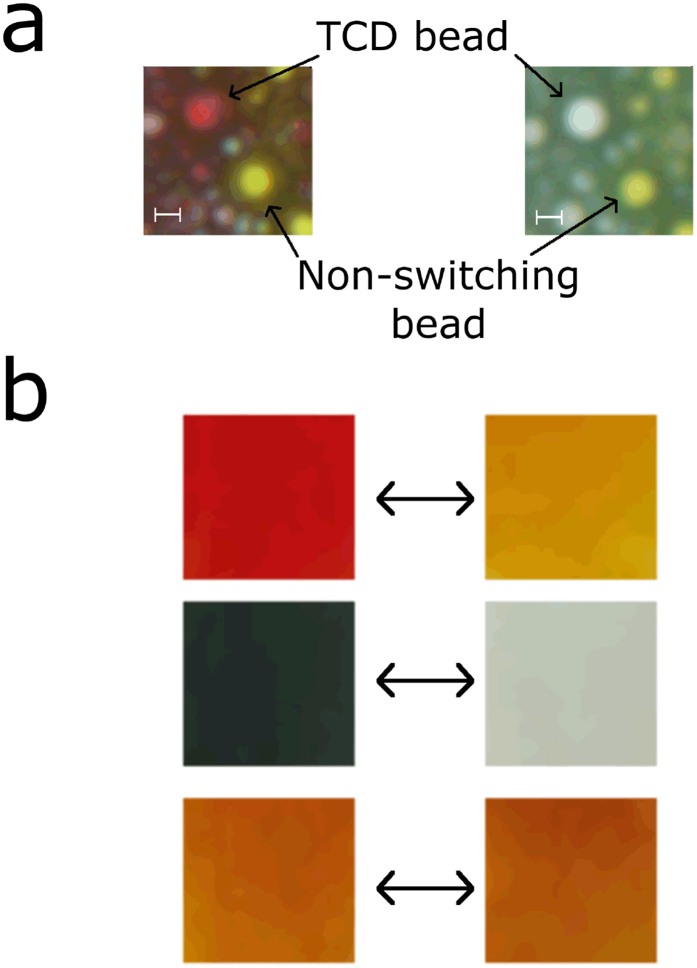
TCD colour switching. (**a**) The orange TCD solution with two components: a red TCD bead with switchable colour and a yellow TCD bead with non-switchable colour. Bar scale, 10 μm. (**b**) Orange (top) and green (middle) colour of TCD solution as well as colour of MNP solution (bottom) before (left) and after (right) switching through heating.

One TCD was green and transitioned to colourless (“dark state”) upon heating (transition temperature of ~31 ± 3 °C) and the other transitioned from orange to yellow at a higher transition temperature (~47 ± 3 °C). The green TCD was made up of thermo-chromic beads in the form of a powder that was subsequently dissolved in a non-polar solvent. The resulting solution transitioned from green to colourless when heated above the transition temperature in a water bath (Fig. 3b). The orange TCD came in the form of a slurry (an aqueous based dispersion of thermochromic beads) that changed colour from orange to yellow when heated above the transition temperature in a water bath (Fig. 3b, top panel). This slurry was composed of two different particles, red thermochromic beads and yellow static beads (Fig. 3a). These two components were able to be separated via centrifugation at a slow speed, as the yellow beads had a higher density than the red beads (see Methods). Several samples were made (orange TCD alone, green TCD alone and MNPs alone) and each sample was observed before and after heating in a water bath. Both TCDs without MNPs changed colour upon heating, whereas the colour of the MNPs remained constant (Fig. 3b, red TCD top, green TCD middle and MNPs alone bottom). Scanning electron microscopy (SEM) images were taken of the orange TCD (showing the red and yellow beads based on apparent density, Fig. 2b), the separated red TCD beads (with a more homogenous bead distribution, Fig. 2c), green TCD beads (Fig. 2d) and the red TCD beads combined with MNPs (red TCD-NP probes, Fig. 2e).

### Testing TCD-NP probes with laser

**Figure 4:**
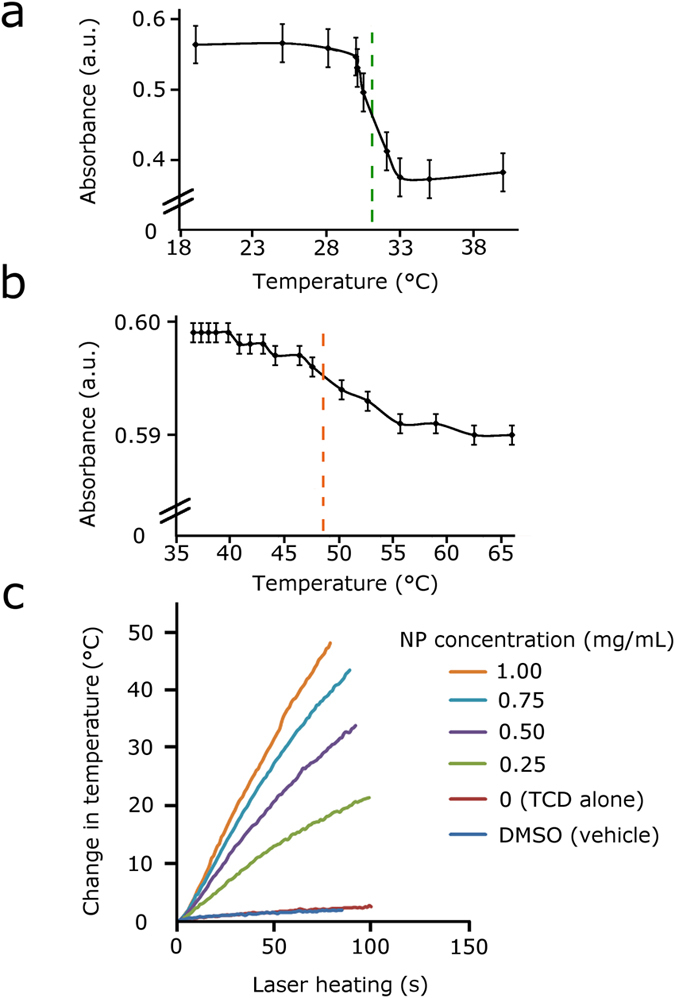
Temperature-dependent absorption and heating kinetics for TCD-NP probes. (**a**) Absorption at wavelength of 630 nm as a function of laser-induced temperature for the green TCD-NP probe (temperature transition range ~31 ± 3 °C). (**b**) Absorption at wavelength of 532 nm as a function of laser-induced temperature for the orange TCD-NP probe (temperature transition range ~47 ± 3 °C). A dotted line estimating the point of inflection as an approximation of the transition temperature threshold for switching. Data represent the standard error for the measurements. (**c**) Heating rate as a function of MNP concentration in orange TCD during laser heating. Laser parameters: wavelength, 805 nm; beam diameter, 5 mm; power, 15 W.

To demonstrate efficient photoswitching of the TCD-NP probes, each were tested in bulk solution. Orange and green TCDs were separately incubated (1–2 hours) with MNPs to create the TCD-NP probes. Each of these probes were then irradiated by laser light at wavelength of 805 nm, and absorption spectra was periodically measured using a table top Ultrospec 3300 pro UV/Visible spectrometer to determine the spectral shifts that were associated with laser heating of the probes (as described in the methods section). Absorption spectra were recorded in the spectral range from 400 nm-1100 nm, and the absorbance at wavelength of 630 nm for the green TCD-NP probe and at 532 nm for the red TCD-NP probe was measured to determine a representative shift in spectra as a function of temperature. The change in temperature was observed by an infrared red (IR) thermometer, which measured the external surface the solution inside a plastic micro-centrifuge tube. Using this data, we were able to obtain a temperature profile (Fig. 4a,b), showing absorption at different temperatures for each of the TCDs. Interestingly, it was observed that the discolouration is stabilized when the temperature reaches a certain threshold and all absorption values remain approximately stable even as the temperature continues to increase (Fig. 4a,b). We observed slow, steady changes in absorbance and temperature when bulk solutions of the probes were heated with the laser. There was a clear transition from the coloured (“light”) state of the solution to the colourless (“dark”) state for the green TCD, as well as a transition between two colours (i.e. orange-yellow) for the orange TCD, as indicated by the approximate point of inflection on the temperature profile graphs (Fig. 4a,b). In Fig. 4c, the temperature change is shown as a function of heating duration for DMSO, TCD, and the TCD-NP samples during 120 s of laser heating to demonstrate the kinetics of colour change in solution for the orange TCD-NP probe. There is no observed change in temperature during heating with the laser for both DMSO and TCD separately, indicating the necessity of MNPs for NIR photoswitching.

**Figure 5:**
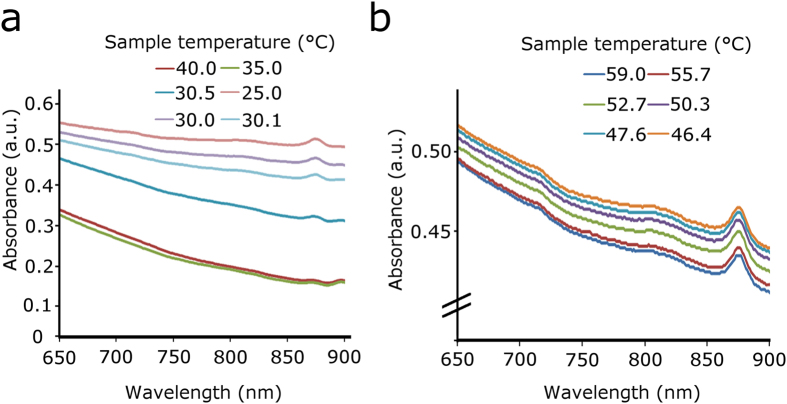
Absorption spectra of TCD-NP probe at different laser-induced temperatures for green (**a**) and orange (**b**) probes.

For both TCDs, a change in absorption spectra was observed at different temperatures when the solutions were heated (via laser illumination, 805 nm), as seen in Fig. 5. The green TCD-NP probe changed from a “light” state to a “dark” state, whereas the orange TCD-NP probe changed from an orange colour to a yellow colour, due to its two colour bead system (Methods). These colour changes corresponded to changes in their absorption spectra. A more exaggerated change was observed for the green TCD (Fig. 5a) when compared to the orange TCD (Fig. 5b) in bulk solution.

### Switching red and orange TCD-NP probes at the single bead level

To enhance spectral differences and observe what happens to the TCD on at the single bead level, the orange TCD was chosen to show the proof-of-principle on the microscopic scale. It became apparent that the orange TCD utilized a two colour bead system, red beads and yellow beads, which caused the bulk solution to have an orange colour when mixed well. We heated orange TCD alone using a heat gun, and using microscopy we found that only the red beads exhibited thermochromic characteristics, changing from red to transparent (Fig. 3a). Thus, the orange TCD changes from orange (red + yellow beads) to yellow (transparent + yellow beads) via this scheme. To obtain a better spectrum by removing the static component (yellow beads)—which could be considered noise— we separated the red TCD beads from the yellow unchanging beads via centrifugation and only used the red TCD beads for the experiments on the microscale to focus solely on the thermochromic aspect of the dye. This enhanced changes in the absorption spectrum dramatically.

**Figure 6:**
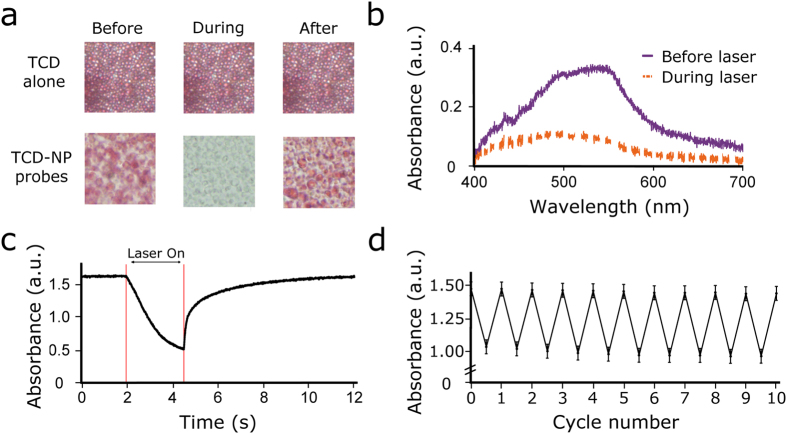
Photoswitching of red TCD-NP probe. (**a**) Images of red TCD alone (top) and red TCD-NP probes (bottom) before, during and after laser heating at wavelength of 805 nm. The time scale is on the order of seconds for the picture taken after laser illumination (see c). (**b**) Spectral data before and during laser heating of the red TCD-NP probes (power ~0.25 W). (**c**) Kinetics of colour change before, during and after laser heating. (**d**) Consistency of switching reversibility of the red TCD-NP probes. Data represent the standard error of the measurements.

**Figure 7:**
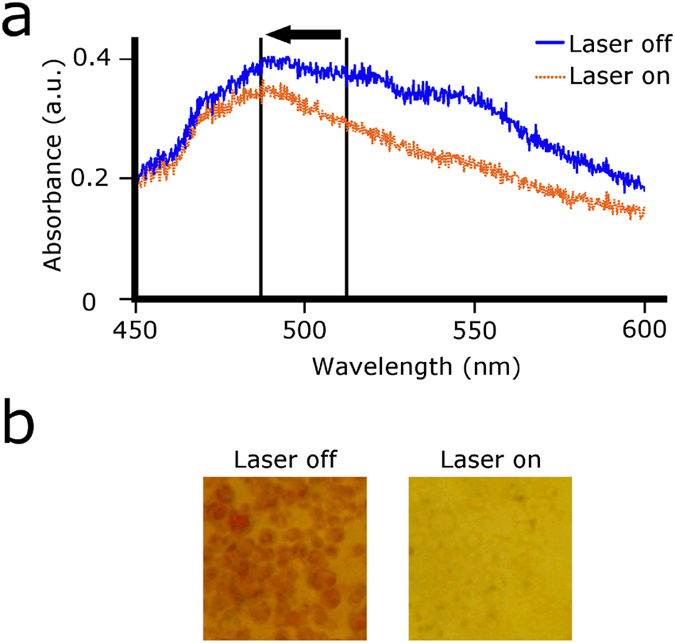
Photoswitching of orange TCD-NP probe. (**a**) Absorption spectra of the orange TCD-NP probe during laser heating at wavelength of 805 nm and power of ~0.5 W. These data demonstrate the slight spectral (blue) shift. (**b**) Visual data demonstrating colour photoswitching at laser parameters indicated in (**a**).

Red TCD-NP probes were studied using a microscopic setup. A defined area was illuminated with 805 nm laser just as was done in bulk solution. Rapid colour/absorption change before and after heating the red TCD-NP probe (Fig. 6a–c) was monitored by a spectrometer connected to the microscope via an optical fibre (Fig. S1). Specifically, we observed the decrease in absorption within milliseconds of laser illumination and a return of colour within milliseconds of laser cessation. A relatively low laser power (down to ~250 mW for dry samples) was needed to heat the solution. It should be noted that the images for the probes (Fig. 6) were taken after the TCD-NP mixture was allowed to dry over several days. Spectral data (Fig. 6b) was taken to confirm what was observed using imaging (Fig. 6a). This change in absorption spectrum was repeatable over many cycles (Fig. 6d) and was not seen in TCD samples that did not contain MNPs (Fig. 6a). The colour change process shown in Fig. 6 can be seen in real time in Supplementary Video 1. In a similar manner, we used the original orange (red beads + yellow beads) TCD to test orange TCD-NP probes at the single bead level in effort to obtain a shift in absorbance from one colour to another upon heating, as opposed to an absorption decrease only. Figure 7a shows the slight spectral shift we observed with this dye. The thermochromic effects were the same as those described above for the red TCD-NP probe, as it was the same red beads that produced this effect.

### Testing TCD and TCD-NP probes in cells

**Figure 8:**
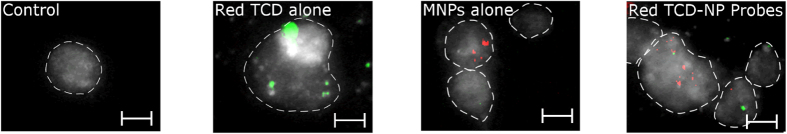
Combined optical and PA images at two wavelength (532 nm and 820 nm) of breast cancer cells (MDA-MB-231) with TCD alone and TCD-NP probes. Four samples: control with no TCD or MNPs; samples incubated overnight with TCD, MNPs or TCD-NP probes. 532 nm PA imaging represented by green overlay and 820 nm PA imaging represented by red overlay. A dotted line has been drawn to highlight the approximate perimeter of the cell. Bar scale, 10 μm.

**Figure 9:**
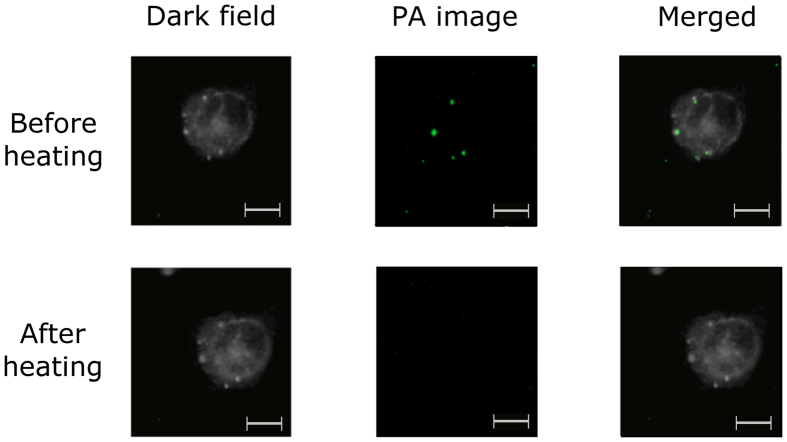
*In vivo* switching of orange TCD-NP probe into cells. The dark field (scattering), PA and combined images of cells (epithelial EA.hy926 cells) were obtained after incubation with red TCD beads. PA images were taken using a laser at 532 nm. Bar scale, 10 μm.

To show the proof-of-concept that our TCD-NP probes could be incorporated into cells and visualized with PA imaging, MDA-MB-231 cells—a breast cancer cell line— were incubated in culture overnight with red TCD alone, red TCD-NP probes, and NPs alone in effort to obtain PA images of the TCD beads and probes inside cells. Using a combination of dark field (scattering, see methods) and PA microscopy, we were able to image MDA cells with incorporated TCD beads (Fig. 8). We were able to distinguish between organelles inside the cell and TCD beads or MNPs, as the TCD beads and MNPs can be seen with PA imaging and the rest of the cell does not produce PA signals (Fig. 8, leftmost panel). To distinguish between TCD beads and MNPs, two colour PA imaging was used: TCD beads were imaged using their dominant absorption at 532 nm (shown as green), and MNPs were imaged using their dominant absorption at 820 nm (shown as red). In Fig. 8, it can be seen that TCD and MNPs are able to associate with a cell when incubated alone. Further, the final panel in Fig. 8 shows a co-localization of the TCD-NP probe within cells. Cells that were not incubated with the TCD beads or TCD-NP probe (i.e. control) did not give off any PA signals, ensuring that any signals obtained in the samples were associated with the TCD beads or TCD-NP probe. There was minor overlap in the 532 nm image, especially in the case of localized clusters of MNPs due to the fact that MNPs have a broad absorption spectrum. To mitigate this effect, the power of the 532 nm laser was reduced to favour signals from the larger TCD beads with dominant absorption at 532 nm relative to the MNPs, allowing the signals to be separated. EA.hy936 cells were also incubated with the red TCD and these cells likewise incorporated the TCD beads into them (Fig. 9). Once we successfully showed that the TCD beads were visualized in cells via PA imaging, we heated the medium with a heat gun and observed a corresponding loss of PA signals from the TCD beads after being heated. We demonstrated this in EA.hy926 cells by taking a PA image before the cells were heated, when the temperature of the medium was room temperature, and after heating the medium with a heat gun to about 49 °C (Fig. 9).

## Discussion

Here we have shown that TCD and MNPs can be combined to create new photoswitchable probes in which the absorption thereof can be modulated using NIR light. We selected MNPs for several reasons: 1) MNPs have a broad absorption spectrum that simplifies the laser choice and allows wavelengths in the NIR range with deeper penetration in bio-tissues (i.e., can be used as universal contrast agents); 2) despite the slight decrease in absorption as wavelength increases, NIR absorption of MNPs is still high enough to provide good PA contrasts with different lasers^28^; 3) MNPs also have added benefits such as their low toxicity (e.g., compared to carbon nanotubes) and some MNPs are approved for clinical application^29^,^30^,^31^ and research^28^; 4) MNPs can be used as multimodal diagnostic contrast agents simultaneously for PA and magnetic resonance imaging; and 5) MNPs can be used as theranostic agents for PT and magnetic treatment of cancer using hyperthermia (through MNP heating). Using MNPs also theoretically provides the system with another strategy of heating (and thus switching) via exposure to a magnetic field^32^.

The effectiveness of the TCD-NP probes for PA methods is heavily dependent on the ability to change their absorption profile using NIR illumination. In particular, these probes demonstrated changes in their absorption when the sample was heated using an 805 nm laser. The change in absorption was most rapidly and drastically seen at the single bead level, likely owing to the different mechanisms of warming of the solution (medium heating in bulk vs. direct heating at the single bead level). We conducted a similar experiment with the orange TCD combined with MNPs to obtain a spectral shift in absorption as opposed to a spectral decrease in absorption (Fig. 7). This shift, however, is not as dramatic as the “light” and “dark” states in which the TCD-NP probe simply lost its absorbance. This is likely due to the relatively small difference between orange and yellow in absorbance and the fact that the TCD is not truly orange, but rather a mixture of red beads and yellow beads, which makes it appear orange. The difference, however, was sufficient to show the proof-of-concept that multicolour probes are feasible using different switching mechanisms.

Though the probes certainly will need to be further studied and enhanced for *in vivo* applications, we cultured cells with the TCD-NP probes and used PA imagining to show the system’s ability to detect absorption change in cell culture. MNP and TCD signals were able to be separated to show each signal alongside one another. We also found that the PA signals that were given from the TCD beads subsequently disappeared after heating the medium (Fig. 9), indicating not only that the cell can take up the TCD beads, but the TCD beads are able to switch *in vivo*. Further studies are required to optimize this system *in vitro* and *in vivo*, assess the toxicity of the TCD-NP probes and explore the use of different NPs such as gold NPs in similar TCD combinations.

The development of a photoswitchable probe that is responsive in the NIR range has the potential to advance the way we approach the study of cell dynamics, offering a non-organic PA contrast agent that can be easily synthesized and tailored to cells or organelles of interest. This concept and platform, generalized to be able to fit the needs of *in vivo* studies, can be used in the future to track bacteria or cancer cells in the bloodstream to study their dynamics and seeding processes. This research also opens up possibilities to enhance magnetic hyperthermia cancer treatments, and the possibility to develop these TCD beads into a micro/nano thermometer. The ability to track various cells using a NIR photoswitchable probe has great potential for deep vessel study and early diagnosis in patients, as NIR light is not heavily attenuated by body tissue components such as water and haemoglobin^12^,^33^. We are confident that the proposed synthesis strategy and first proof-of-concept probes serve as a basis for synthesizing many other NIR probes with different dye and NPs for assessing cells in deep bio-tissue.

## Methods

### Nanoparticles and dye

Two different TCDs were obtained from LCRHallcrest (Glenview, IL)^27^. These thermochromic leuco dyes were composed of a dye and developer (BPA) encapsulated in melamine formaldehyde. The dye and developer are in an ordered structure below the melting point of the solvent and released from one another after the melting point of the solvent is surpassed, which corresponded to its coloured and colourless states, respectively. One dye transitioned from green to transparent when the threshold temperature of 31 ± 3 °C was surpassed and the other transitioned from orange to yellow when the threshold temperature of 47 ± 3 °C was surpassed. The green TCD came in the form of dry power which was subsequently dissolved in DMSO. The mixture was then homogenized using a sonicator (Fisher Scientific, FS20D) for 5 minutes. The orange TCD came in the form of a slurry (an aqueous based dispersion of the thermochromic microparticles). Upon further investigation, it was observed that the orange TCD utilized a two component system to change from orange to yellow, employing red thermochromic beads that transition from red to transparent and yellow beads that remained static regardless of temperature, causing the overall solution to transition from an orange colour (red beads + yellow beads) to a yellow colour (transparent beads + yellow beads).

Magnetic iron oxide (II, III) nanoparticles, with average size of 20 nm and concentration of 5 mg/ml dispersed in water, were purchased from Sigma-Aldrich (St Louis, MO) and used to make the TCD-NP probes. Different solutions of TCDs were thoroughly mixed in an Eppendorf tube via pipetting, creating the TCD-NP probes. Experiments done on the microscale utilized a ratio of 1:4 TCD to iron oxide nanoparticles to make the TCD-NP probes.

### Red TCD sample preparation

The orange TCD was observed to be comprised of two components, red thermochromic beads and yellow static beads, which made the solution appear orange at a distance. However, under microscopy, both colours were distinctly recognizable. To focus on the mechanism of absorption change, the active red beads were separated from the static yellow beads using a small table centrifuge (Qualitron Inc.) at a low speed. Experiments done on the microscale used these separated red TCD beads alone to ascertain a better spectra. For this reason, this TCD is referred to as orange (red + yellow) TCD when used in bulk solution and red TCD when observed on the microscale.

### Heating and spectral analysis of TCDs alone

To observe the colour/absorption changes of each TCD alone strictly due to temperature change, UV-VIS spectroscopy was carried out with a UV-3600 spectrometer from Shimadzu on samples with different concentrations of TCD heated and cooled using water. Samples of each TCD alone, MNPs alone and different concentration of TCD-NP probes were cooled below their respective transition temperature in ice water and then heated above this temperature in a water bath. The onset and ending of colour/absorption change was measured by using the spectrometer mentioned above during the heating and cooling cycles.

To heat the TCD beads on a microscope slide and obtain data from the single bead level, a Master Appliance ECOHEAT heat gun (model EC-100K) was used to elevate the temperature well above the transition point for the each TCD. In each heating-cooling cycle for the red TCD, the sample was heated for 10 seconds and allowed to cool for two minutes while spectral data was recorded. Images were taken on an Olympus microscope (IX81) and spectral data was recorded using an Ocean Optics USB4000 spectrometer which recorded spectral data from a defined area in the view of the microscope via an optical fibre that connected the spectrometer to the optical lens set up of the microscope (Fig. S1).

### Laser heating and spectroscopy analysis of TCD-NP probes

We tested the heating ability of the TCD-NP probes using a NIR Diomed laser 25 with a wavelength of 805 nm from Dotmed World. The beam diameter was 5 mm. The laser diode was set to deliver an output power of 15 W on continuous wave mode through the collimator and was located 10 mm above the sample, perpendicular to the sample. An Ultrospec 3300 pro UV/Visible spectrometer from Amersham Biosciences (400 nm–1100 nm) was used for measurements of spectral absorption of the all samples during heating and cooling cycles. An IR thermometer camera from PTM0.1 was used to measure the temperature of the samples, and an IR FLIR camera (FLIR systems, Thermovision A40M) placed in front of sample at a distance of 100 cm was used to measure the external surface of the samples. The solution was exposed to three minutes of laser irradiation and allowed to cool for three minutes. IR images were taken every 10 seconds during both heating and cooling cycles. The kinetics of laser heating were obtained from spectral data that was taken under the conditions of 15 W of continuous wave 805 nm laser light in one second intervals every two seconds (one second laser on, one second laser off). The samples were illuminated in this way for 85 seconds and the absorption spectrum (from 400–1100 nm) was recorded before heating, and at several temperatures as the solution was cooling down after heating.

To compare the results found in heating the bulk solution with microscopic results, slides were made of concentrated red TCD (184.5 mg/mL) alone as a control and with the concentrated red TCD solution mixed with MNPs (5 mg/mL) in a 1:4 ratio, respectively, to make the TCD-NP probe. Each was then illuminated with the near-infrared diomed laser at a wavelength of 805 nm at various intensities ranging from ~0.25 W to ~2 W, respectively. Spectral data was recorded using the same Ocean Optics USB4000 spectrometer, which recorded spectral data from a defined area in the view of the microscope, as described in the previous section (Fig. S1).

### Cell culture and imaging

MDA-MB-231 (ATCC HTB-26) adenocarcinoma cells were cultured in DMEM media using the Nunc Lab-Tek II Chamber Slide System to grow the cells on the slides that would later be used for analysis. This cell line was used to demonstrate that live cells would take up the TCD. EA.hy926 (ATCC CRL-2922) epithelial cells were also incubated and analysed in the same manner as the MDA-MB-231 cells. Cells were allowed to grow until they were confluent and then they were incubated with the red TCD and TCD-NP probes overnight. After incubation, the chamber was washed 3–5 times and refilled with PBS to carry out optical and PA microscopy. To create the optical images (i.e., not PA), we used dark field imaging using the detection of scattered light only. The light direction used to obtain the image was not perpendicular to the sample, but rather positioned at an angle so that scattered light entered the micro-objective (Fig. S2). Therefore, each image was obtained using scattered light only (as opposed to transmission). For this reason, the cell imaging technique used here is referred to as “dark field (scattering)” imaging.

### Photoacoustic imaging

PA imaging was taken to observe red TCD beads *in vivo* using a custom laser scanning PA microscope based on an Olympux IX81 inverted microscope platform. Galvo mirrors (6215H, Cambridge Technologies, Lexington, MA) were used to steer 532 nm and 820 nm laser beams (10 kHz pulse repetition rate) coupled to the microscope using single mode optical fibres. The focal area of the transducer (V316, 20 MHz, 12 m focal distance, Olympus-NDT Inc.) limited the imaging area to 150 μm. Cells were incubated in the chamber slides as mentioned above and the chamber walls were extended to accommodate the distance needed from the transducer to the area of interest. XYZ adjustment of the transducer position was obtained using a custom holder that positioned the transducer over the sample. Signals from transducer were amplified (5662B, Panametrics) and recorded by PC equipped with a high-speed digitizer (PCI-5124, 12-bit card, 128 MB of memory, National Instruments, Austin, TX). A digital waveform generator (DG4062, Rigol, Beijun, China) gave control over the mirrors and synchronization of the system. The signals generated from this scanning were then translated into an image using custom made software based on the LabView platform.

## Additional Information

**How to cite this article**: Harrington, W. *et al*. Photoswitchable non-fluorescent thermochromic dye-nanoparticle hybrid probes. *Sci. Rep.* **6**, 36417; doi: 10.1038/srep36417 (2016).

**Publisher’s note**: Springer Nature remains neutral with regard to jurisdictional claims in published maps and institutional affiliations.

## Supplementary Material

Supplementary Video 1

Supplementary Information
