# Supplementary material for: Photoswitchable non-fluorescent thermochromic dye-nanoparticle hybrid probes
Source: Sci Rep. 2016 Nov 8;6:36417. doi: 10.1038/srep36417 (PMC5099817; doi:10.1038/srep36417)
Supplement: Supplementary Information [file srep36417-s2.pdf]

Photoswitchable non-fluorescent thermochromic dye-nanoparticle hybrid probes

Walter Harrington†″*, Mwafaq R. Haji‡″, Ekaterina I. Galanzha†, Dmitry A. Nedosekin†, Zeid A. Nima‡, Fumiya Watanabe‡, Anindya Ghosh*, Alexandru S. Biris‡[+], and Vladimir P. Zharov†[+]

†Arkansas Nanomedicine Center, University of Arkansas for Medical Sciences, Little Rock, AR

‡Center for Integrative Nanotechnology Sciences, University of Arkansas at Little Rock, Little Rock AR

*, Chemistry Department, University of Arkansas at Little Rock, Little Rock AR

″These authors have contributed equally to the work and both should be considered first authors.

[+]Principle Investigator

*Corresponding Author Contact: wnharrington@uams.edu

Supplementary information

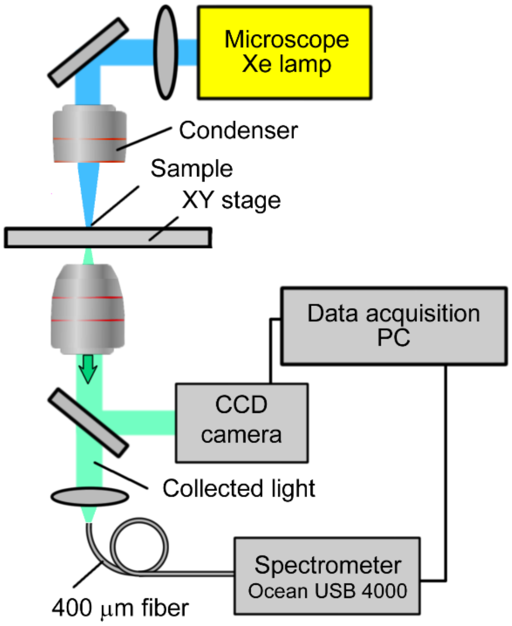

Supplementary Figure S1. Microscope tethered absorbance spectroscopy schematic. Schematic depiction of the set up used to simultaneously obtain optical images and absorbance spectrums.

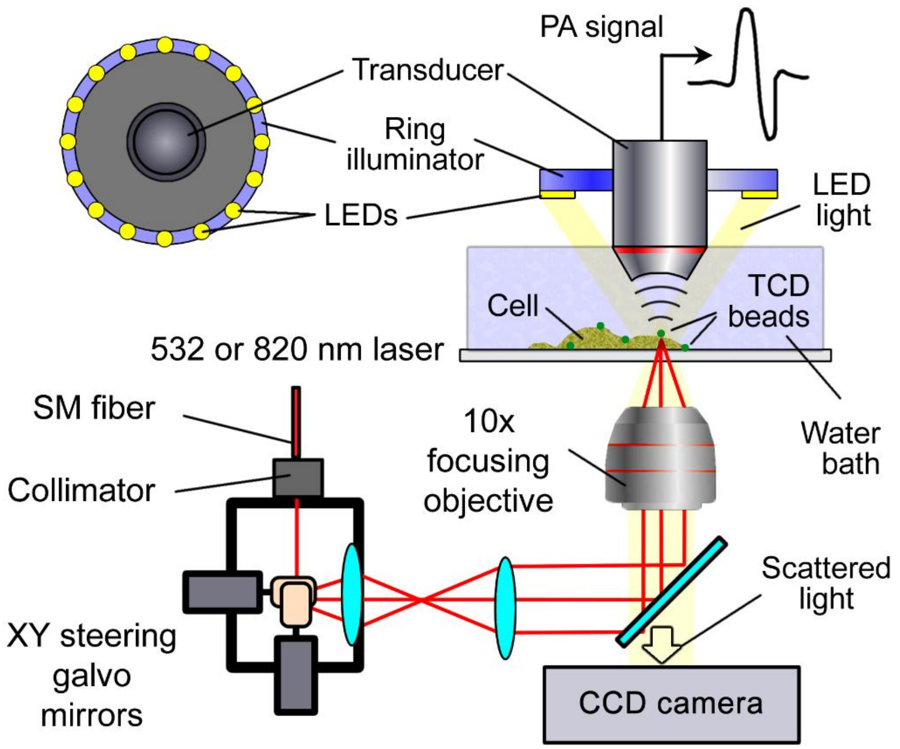

Supplementary Figure S2. Schematics of photoacoustic and optical imaging. Schematic detailing the set up used to obtain optical and photoacoustic imgages of cells with TCDs and TCD-NP probes.

Supplementary video 1. Real-time photoswitching of red TCD-NP probe. Microscope video of TCD-NP probed during laser illumination. Laser parameters: wavelength, 805 nm; power, ~0.25W.
